# Corticotropin releasing hormone is present in the feline placenta and maternal serum

**DOI:** 10.3389/fendo.2023.1132743

**Published:** 2023-04-14

**Authors:** Madison Watt, Pardis Mohammadzadeh, Emma Pinsinski, Fiona K. Hollinshead, Gerrit J. Bouma

**Affiliations:** ^1^ Department of Biomedical Sciences, Colorado State University, Fort Collins, CO, United States; ^2^ Department of Clinical Sciences, Colorado State University, Fort Collins, CO, United States

**Keywords:** CRH, cat, pregnancy, biomarker, placenta

## Abstract

**Background:**

In women, placental corticotropin releasing hormone (CRH) can be detected in maternal blood throughout pregnancy and is important in the regulation of the timing of parturition. However, its role in other mammalian species is unclear. In fact, very little is known about the presence and localization of CRH in placentas other than human. In this study we report for the first time the presence of CRH in feline placenta and maternal serum.

**Methods:**

Presence of CRH mRNA and protein was assessed using RT-PCR and Western blot, respectively, in at term domestic cat placentas opportunistically obtained at a local animal shelter and spay clinic. In addition, CRH localization within the placenta was demonstrated *via* immunohistochemistry. Finally, presence of CRH in maternal blood from early (¾21 days) and mid (25-35 days) stages of pregnancy was investigated by ELISA.

**Results:**

CRH mRNA and protein were detected in feline placentas, and localized to larger decidual cells and fetal trophoblast cells, including the binucleate cells. CRH was detectable in maternal blood collected from early-stage pregnancies, and amounts significantly increased in mid-gestation samples.

**Conclusion:**

This is the first report on the presence and localization of CRH in the feline placenta, and its increase in maternal serum during the first half of pregnancy. These data lay the foundation for future studies to determine if CRH can be used as potential novel marker for early pregnancy diagnosis, determination, and monitoring in felids, and could greatly increase efficiency and success in zoo breeding programs utilizing artificial reproductive technologies for endangered feline species.

## Introduction

Corticotropin releasing factor, from here on will be referred to as corticotropin releasing hormone (CRH), is a peptide hormone released by hypothalamic neurons in the paraventricular nucleus that activates the hypothalamic-pituitary-adrenal (HPA) axis. Circadian rhythm is the main regulator of CRH release and is necessary for maintaining normal homeostasis for example, in response to many exogenous and endogenous stimuli including stress. CRH leads to the release of adrenocorticotropic hormone (ACTH) from the anterior pituitary gland, which in turn acts on the adrenal cortex to stimulate the release of cortisol. Furthermore, during pregnancy, cortisol derived from the maternal adrenal gland plays a central role in fetal growth and organ maturation. Because of its immunomodulatory function, cortisol also plays an important role in establishment and maintenance of pregnancy by inhibiting immune rejection responses around the time of implantation. During pregnancy cortisol together with progestins play an important role in the regulation of maternal immune tolerance. A fine balance between cortisol and progesterone signaling is necessary for the maintenance of pregnancy in humans through the regulation of endometrial receptivity, uterine quiescence, and placental vascularization (reviewed in 1).

In addition to the hypothalamus, CRH is present and released from the placenta in humans and non-human primates with levels low at the start of pregnancy but increasing exponentially and peaking during labor (2–4). It’s presence in the placenta and maternal circulation during pregnancy has not been reported in domestic species. Immunoreactivity for CRH was detected in the hormone secreting syncytiotrophoblast layer of the placenta and maternal decidua in humans (5, 6). The synthesis of CRH by the primate placenta leads to increased cortisol levels in the fetus thus promoting lung maturation and synthesis of surfactant and proteins required for survival after birth. In late pregnancy, CRH is involved in stimulation of myometrial stretching to increase uterine contractility and facilitate parturition (3). Finally, CRH is thought to act on CRH receptors in the placenta to activate the fetal pituitary-adrenal axis and promoting secretion of precursors necessary for placental estrogen synthesis and initiation of parturition in all species (reviewed in 7).

The significant increase in human maternal CRH levels near term has led to investigations into its use as a biomarker of preterm/premature delivery or pregnancy complications. Measurement of plasma levels of CRH is used as a tool by clinicians to monitor pregnancy complications in women as significant elevations are associated with pre-term/premature neonatal/fetal delivery (3, 8, 9). Alternatively, considering its detection in the placenta and presence in maternal serum during the first trimester of pregnancy would also allow for its use as an early biomarker for pregnancy (4, 10). A molecular biomarker of early pregnancy would greatly benefit endangered feline breeding programs as it would facilitate increased efficiency and success in breeding programs utilizing artificial reproductive technologies.

Current methods of pregnancy diagnosis in felids includes transabdominal ultrasound, abdominal palpation, radiography and detection of relaxin in serum and urine. Transabdominal ultrasound can reliably detect pregnancy in domestic cats from day 25 of gestation (Davidson AP, 2020, MERCK Manual, Veterinary Manual) but with higher quality ultrasound equipment and operator skill, gestational sacs can be detected in the domestic cat on day 10 after mating and the embryo can be detected as a hyperechogenic spot in the gestational sac from day 14 after mating (11). However, this early ultrasonographic detection of pregnancy is not possible in most wild feline species due to a significantly larger body size to the domestic cat and practical issues involving safety and restraint. Abdominal palpation of fetal vesicles in the uterus is possible to perform in domestic cats from days 21-25 after mating (12) but is not reliable and not a practical option for large, exotic felids. Abdominal radiography performed after day 38-40 when calcification of the fetal bones has occurred is a reliable method of pregnancy diagnosis but only in late gestation (12).

Although the queen is an induced ovulator, progesterone is not a useful marker for pregnancy as ovulation in the queen can also occur spontaneously with a reported incidence in domestic cats ranging from 30 to 87% (13–15). A pseudopregnancy period occurs after spontaneous ovulation with progesterone detectable for up to 40 days. Detection of the placentally secreted hormone, relaxin, has been used as a marker for early pregnancy in both domestic and wild cats. Relaxin can be first detected in both the urine and serum of pregnant cats between days 21 and 28 of gestation using radioimmunoassay (16). Efforts were put into different assay methods to try and detect relaxin in either the urine or serum earlier in pregnancy (17). Unfortunately, no method trialed resulted in the detection of relaxin earlier in pregnancy (< 20 days) which would be beneficial to many captive feline wildlife breeding programs that implement complex artificial reproductive technologies such as Laparoscopic Oviductal Embryo Transfer and Artificial Insemination (18). Currently molecular biomarkers for detection of pregnancy within the first 3 weeks of pregnancy does not exist for felids. Such a biomarker to detect pregnancy early would be of significant benefit to captive zoo breeding programs involving endangered exotic feline species where reproductive efficiency is poor and the implementation of novel artificial reproductive technologies (ART) is necessary to try and propagate these endangered species. Detection of early pregnancy would facilitate more effective implementation of specific ARTs in these rare breeding programs.

The overall goals of this study were to determine if CRH; i) is present and produced by the feline placenta, ii) can be detected in serum of pregnant felines/queens, and if CRH is present, iii) can be detected early in pregnancy and increases in maternal serum during pregnancy. Little is known about the presence and localization of CRH in species other than human placentas. Not only would this expand our current understanding on the possible role of CRH in different placental types as it relates to pregnancy and/or parturition, but in feline species this could provide for future opportunities to identify a novel marker for early pregnancy diagnosis, determination, and monitoring.

## Materials and methods

**Figure 1:**
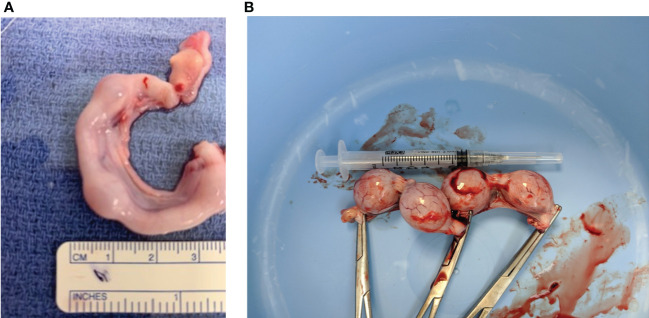
Representative images of two early stage feline embryo collections. **(A)** Right uterine horn indicating embryos less than ~3 weeks of gestation. **(B)** Four fetuses (~25 mm) collected from of a ~21 day pregnant feline. (Dr. Shauna O”Meara, BVSc, “Stage of Cat Pregnancy”).

Post-partum domestic cat placental tissues were obtained opportunistically from a local animal shelter and spay clinic. Sections of the zone of attachment containing the labyrinth layer of the placenta were removed and snap frozen or fixed in 4% paraformaldehyde for RNA and immunohistochemical analysis, respectively. Blood samples also were opportunistically obtained from remaining blood taken for pre-anaesthetic profiles prior to ovariohysterectomy of pregnant and non -pregnant queens from the same local animal shelter and spay clinic. Blood samples from queens in early (¾21 days) and mid (25-35 days) stages of pregnancy (**Figure 1**) were analyzed for CRH presence and concentration by ELISA. Staging of fetal gestation was performed using fetal morphology measurements (19).

### RNA isolation

Total RNA was isolated from postpartum placental tissue samples (the zonary region) using Qiagen RNeasy mini kit (74104) according to the manufacturer’s instructions. Briefly, approximately 30 mg of frozen tissue was homogenized in RLT Lysis buffer, processed for RNA isolation, and eluted in 30μl of DNAse/RNAse free water. RNA purity and concentration was analyzed using a NanoDrop ND-1000 UV-Vis spectrophotometer. All samples used for subsequent analysis had a 260/280 absorbance ratio between 1.8 and 2.2.

### Reverse transcription

Total RNA was reverse transcribed into cDNA using the Bio-Rad iScript cDNA synthesis kit (1708891) according to the manufacturer’s instructions. Briefly, 4μl iScript reaction mix, 1μl reverse transcriptase, and 1μl (500-1000ng) cDNA were combined. The placental tissue samples were placed in a thermocycler with an initial step at 25°C for 5 minutes for priming, followed by 46°C for 20 minutes for reverse transcription, and 95°C for 1 minute for reverse transcriptase inactivation. The samples were stored in the -20°C freezer overnight.

### Polymerase chain reaction and gel electrophoresis

To confirm the presence of CRH mRNA in postpartum placental tissue, forward and reverse primers were designed. Briefly, the feline CRH sequence was obtained in Ensembl (https://uswest.ensembl.org/index.html), and Primer3 (https://bioinfo.ut.ee/primer3-0.4.0/) was used to design primers. Primer sequences are provided in **Table 1** and are deigned to amplify ~190bp sequence fragment of the CRH transcript. The PCR protocol includes an initial denaturation step at 94°C, followed by an annealing step at 60°C, and an extending step at 72°C. These steps were repeated 40 times followed by a terminal hold at 4°C. The samples were resolved and visualized on a 2% agarose gel containing ethidium bromide and included a GoldBio 100bp PLUS DNA ladder (Gold Biotechnology, D003-500). The gel was run at 125V for 45 minutes and visualized using a UVP Benchtop 2UV transilluminator.

**Table 1:** Primer sequences used to amplify a fragment of feline CRH transcript.

Name	Sequence 5’ to 3’
CRH-Forward	CAACTTTTTCCGCGCGTTG
CRH-Reverse	TTCTCGGAGGAGGTGGAAGG

The expected amplicon size is 190bp.

### Protein isolation and concentration

A small fragment of frozen post- partum placental tissue (the zone of attachment containing the labyrinth layer) was collected and combined with 300 μL RIPA lysis buffer (ThermoFisher Scientific, 89901), and 10 μl of protease and phosphatase inhibitor and EDTA cocktail (TermoFisher Scientific, 78429) were added to each sample. After homogenization samples were sonicated and centrifuged at 10,000 rpm at 4°C for 10 minutes. The pellet was discarded and the supernatant was added to a new tube with 3 μL PMSF (Roche, 39212624). A BCA assay was conducted, and protein concentrations were determined using a Pierce™ Protein Assay Kit (ThermoFisher Scientific, 23227) and a Fluostar Omega (BMG Labtech) microplate reader equipped with Omega software.

### Western blot analysis

Placental protein samples from post -partum placentas were combined with 6x loading buffer and vortexed and spun down for 15 seconds each. Samples were boiled for 10 minutes, and 50μl containing 30μg of total protein sample was resolved on a 10% Mini-PROTEAN TGX™ Precast Protein Gel (BioRAD, 4561034), which included a Precision Plus Protein WesternC™ ladder (BioRAD, 1610376), at 130V and run for 35 minutes at 4°C. Protein samples were transferred onto a PVDF membrane (ThermoScientific, 88518) at 100V for 2 hours at 4°C. Following 3 washes (5 minutes each) in 1X TBST, the membrane was blocked using 5% powdered milk for 1 hour at room temperature. After blocking non-specific binding sites, membranes were incubated with anti-human CRF rabbit polyclonal antibody (Invitrogen, PA5-102356) at a 1:2000 dilution on a shaker overnight at 4°C. As a negative control, 10% rabbit serum was used without CRH antibody.

The following day, PVDF membranes were washed with 1x TBST (3x, 5 minutes each), and incubated with goat anti-rabbit IgG HRP conjugated antibody **(**1:3000, Abcam ab6721) and Precision Protein StrepTactin-HRP conjugate (1:5000; BioRAD, 1610380) diluted in 5% milk for 1 hour at room temperature. Following 3 washes in 1x TBST for 5 minutes each, membranes were incubated in SuperSignal™ West Dura Extended Duration Substrate (ThermFisher Scientific, 34075) for 5 minutes. The membrane was then visualized and imaged using a BioRAD ChemiDoc MP Image System.

### Immunohistochemistry

Immunohistochemistry was carried out on post -partum and early/mid pregnancy placental tissue (zone of attachment) using paraffin-embedded, 5μm term placental feline tissue sections. Tissue de-waxing and rehydration was performed by two five-minute washes in CitriSolv (Decon Labs, 1601), two five-minute washes in 100% ethanol and four five-minute washes in 90%, 70%, 50% and 30% ethanol and finally deionized water for two minutes. After rehydration, the tissue sections were microwaved for 20 minutes in citric acid-based Antigen Unmasking Solution (pH 6.0; Vector Laboratories, H-3300-250). After washing in 1x PBS for five minutes, the samples were incubated in a humidity chamber with 3% hydrogen peroxide each for twenty minutes. To prevent nonspecific binding, tissue sections were incubated for thirty minutes with Blocking Serum (Normal Serum) from the Vectastain ABC Kit Peroxidase (Vector Laboratories, PK-6101). After blocking, tissue sections were incubated with anti-human CRF rabbit polyclonal antibody (Invitrogen, PA5-102356) at 1:20 diluted in Blocking Serum overnight in a humidified chamber at 4°C. The following day, sections were washed in 1x PBS for five minutes, and incubated for thirty minutes with biotinylated secondary antibody solution provided in the Vectastain ABC kit. After a subsequent thirty minute incubation with Vectastain ABC Reagent, the slides were washed in 1x PBS for five minutes, and incubated with DAB Substrate (Vector Laboratories, SK-4100) for 3 minutes, and sections were mounted in Vectashield Antifade Mounting Medium (Vector Laboratories, H-1500). The slides were then viewed and photographed using a brightfield microscope to identify CRH localization in term placental feline tissue.

### ELISA

Serum was collected from two non-pregnant, two early pregnant and two mid-pregnant cats (as determined by fetal morphological evaluation, see above) have been used to detect CRH. The ELISA test (Cloud-Clone Corp., CEA835Hu) has a detection range between 12.35-1,000 pg/ml and a sensitivity of 5.19 pg/ml and was done in duplicates (n=2) for all samples according to the manufacture’s instruction. Thereafter, data was analyzed *via* Microsoft Excel 365 and Prism 9 using One-Way ANOVA test followed by Tukey’s post- test. Data is presented as mean ± SEM and p ≤ 0.05 was considered statistically significant.

## Results

### Presence of CRH in the feline placenta

**Figure 2:**
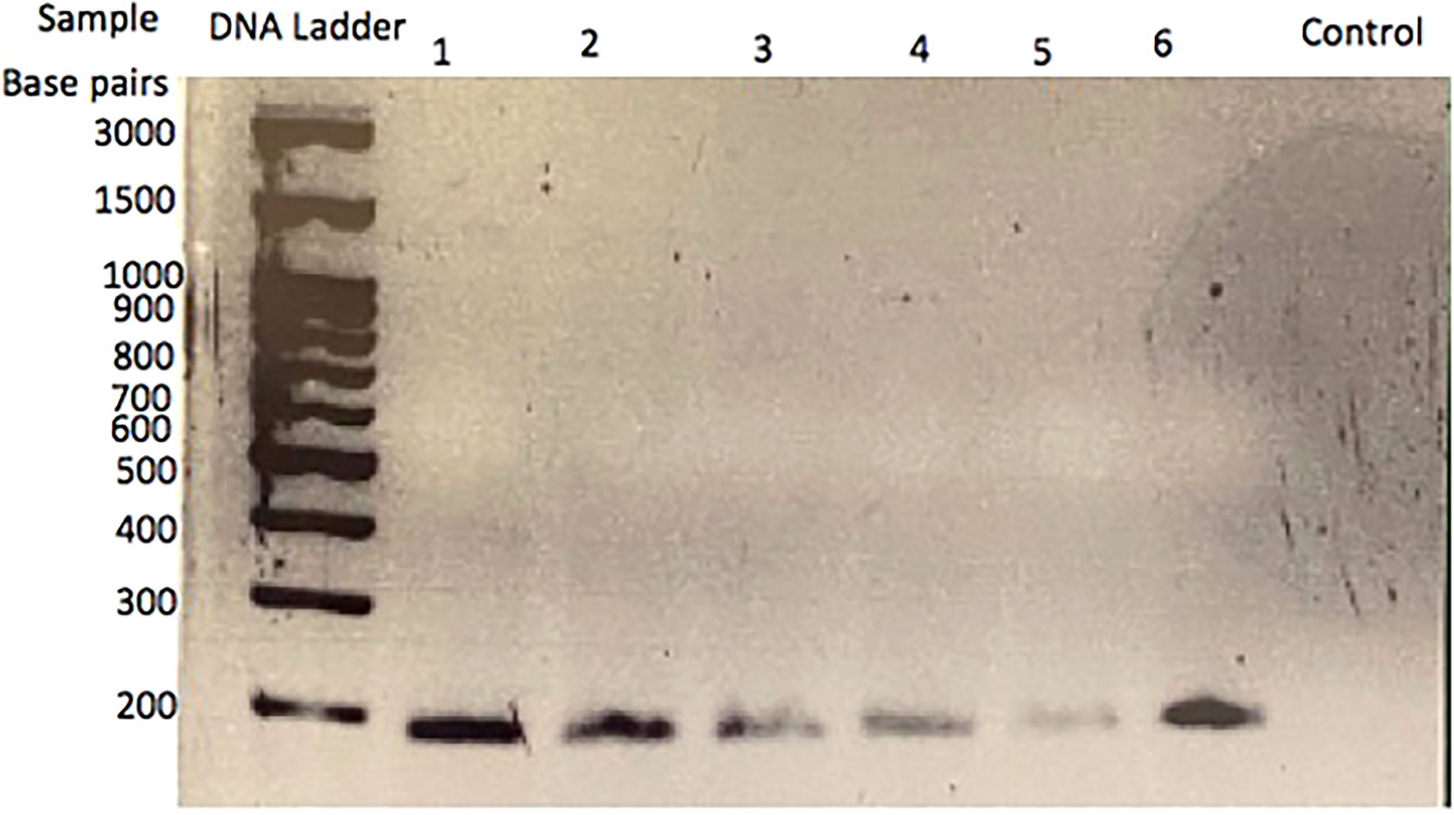
CRH transcript in cat placenta. RT-PCR analysis and gel electrophoresis reveal an amplified cDNA fragment of ~200 base pairs in three different placental tissue samples. Lanes 1 and 2 are sample 1; lanes 3 and 4 are sample 2; lanes 5 and 6 are sample 3. The control lane is a no template, H2O control.

**Figure 3:**
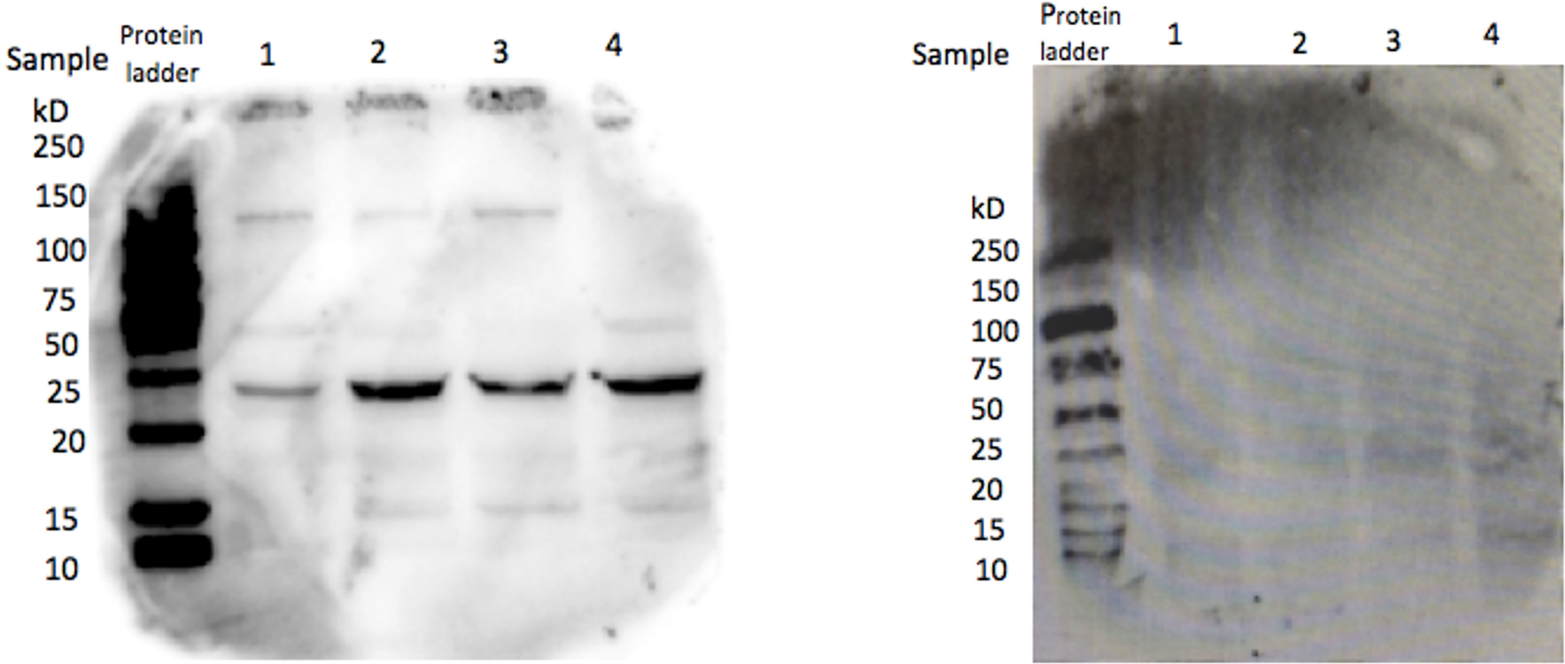
CRH protein in cat placental tissue. Western Blot analysis revealed the presence of a ~22 kDa protein in four placental tissue samples, consistent with the size of CRH preprohormone (left), whereas serum without primary antibody served as a negative control (right).

To determine if CRH is present in the feline placenta, total RNA was isolated from 3 different post-partum placental tissues, reversed transcribed and PCR amplified using primers designed against a fragment of the feline CRH transcript. **Figure 2** indicates the presence of ~190bp cDNA band in placental tissue samples from 3 different cats. Furthermore, Western blot analysis on isolated protein from feline placentas revealed the presence of an ~23kDa sized band, consisted with the size of CRH preprohormone (**Figure 3**).

### CRH localization in the feline placenta

**Figure 4:**
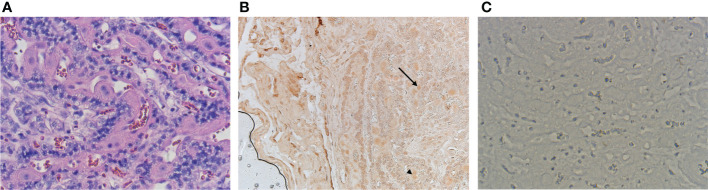
CRH localization in the labyrinth layer of a zonary section of the feline placenta. H&E staining of the labyrinth layer of post-partum placental tissue (**A**). Staining for CRH is observed in fetal trophoblast cells (arrow), including binucleate cells (**B**; arrowhead). No staining is observed when the primary antibody is omitted (**C**). Images were taken at 20X.

In order to determine the cellular localization of CRH within the zone of attachment of the placenta, immunohistochemistry was performed. As indicated in **Figure 4**, positive staining for CRH was detected in larger decidual cells and fetal trophoblast cells, including the binucleate cells of feline placental tissue.

### Presence of CRH in pregnant feline maternal serum

**Figure 5:**
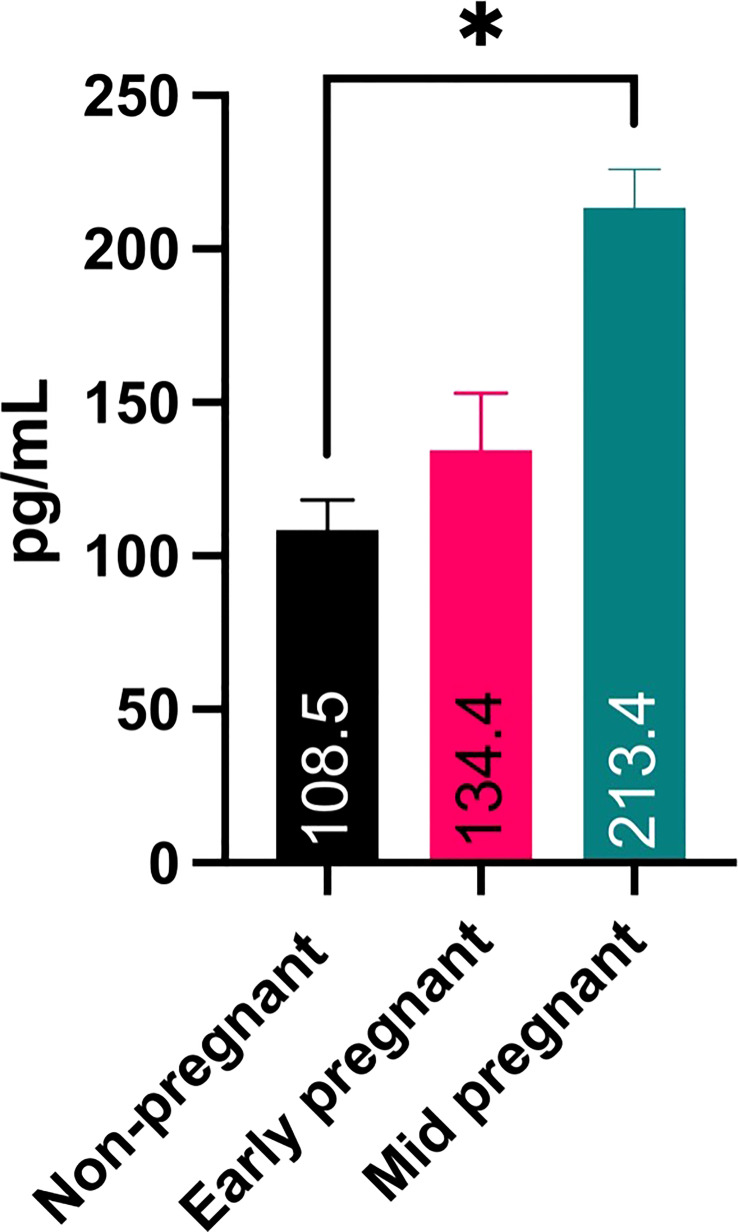
ELISA analysis for CRH in maternal serum samples from early- and mid-pregnant and non-pregnant queens. Sample number is 2 for all three stages, and each were run in duplicate. * P ¾ 0.05.

Demonstrating the presence of CRH transcript and protein in the placenta, we next wanted to determine if CRH is secreted into and detectable in maternal serum during pregnancy. To this end, an ELISA specific for human CRH was used. Results in **Figure 5** reveal that CRH was present in feline maternal serum, and nearly doubled (P < 0.05) from early to mid- pregnancy.

## Discussion

The goal of this study was to determine if CRH is present in the feline placenta and maternal circulation, and if maternal systemic blood levels of CRH increase during early gestation. We opportunistically were able to obtain domestic feline placentas and maternal serum from a local cat shelter and spay clinic. Using primers designed to amplify feline CRH we demonstrated for the first time that CRH mRNA in a species other than human or rodent, and also is present in feline placental tissue and maternal blood. This primer set was designed to amplify CRH in cats and dogs (data not shown). Using a polyclonal antibody we detected an ~22 kDa size protein which corresponds to the predicted size for CRH preprohormone (CRH prohormone is ~19 kDa; CRH mature hormone is ~5 kDa). Additionally, immunohistochemistry revealed CRH positive cells in the fetal trophoblast cells, including binucleate cells. Finally, ELISA analysis on maternal feline serum indicated that CRH levels nearly doubled from 108.5 pg/ml non-pregnant to 213.4 pg/ml mid pregnancy serum levels. CRH in serum from non-pregnant felines likely originate from peripheral tissues, including ovaries, endometrial glands, liver, stomach, intestine, pancreas, lungs, adrenal glands, and inflammatory sites (reviewed in 20–22). In non-pregnant women CRH levels are low and variable ranging from ~6 to ~150 pg/ml (2, 4, 23, 24).

This is the first report not only of the detectable presence of CRH in placenta, but also in maternal serum of pregnant queens. Mid-pregnancy levels of CRH in feline serum were similar to the concentration reported in women during early third trimester of pregnancy (~270 pg/ml; 2). In our study, CRH was detected in postpartum placentas of queens, suggesting CRH likely would have been detected in maternal serum throughout pregnancy. However, collection of late gestation and at term blood samples was not possible, therefore it is unclear if CRH levels in the cat would continue to increase similar to humans, higher primates and sheep. Future studies are required to confirm increasing levels of maternal CRH in pregnant queens. The early detection of CRH in maternal serum and its increase during the first half of pregnancy suggests a potential role for CRH in implantation and fetal development similar to humans and higher primates.

Other than in humans and primates, little is known about the presence of CRH in the placenta. CRH has been detected in umbilical venous blood in sheep late in gestation and rises during the final days before delivery (25), however these do not appear to be the result of tonic placental secretion (26). CRH mRNA has been detected in human and higher (Anthropoid) primate placentas, but was not found to be present in guinea pig, rat and lemur placentas (27). However, similar to our findings in felines more recent studies indicate that in rats CRH mRNA and protein were detected in placental tissue *via* qPCR and Western blot, respectively (28). Maternal CRH levels in pregnant rats did not change with increased gestation length and remained ~3 ng/ml from mid- until late-pregnancy. Furthermore, CRH mRNA is also present in mouse placentas from day 12 of gestation but not near term at day 18 (29). These findings suggests that in mice and rats placental CRH plays a role during implantation and early pregnancy establishment and/or maintenance. This is further highlighted by the observation that leukemia inhibitory factor (LIF), a cytokine necessary for implantation (30), upregulates CRH in cultured mouse trophoblast cells through activation of the PI3K/AKT signaling pathway (31). However contrary to humans where CRH plays a role in the establishment and maintenance of pregnancy and also timing of parturition, this appears not to be the case in rats and mice.

The early detection of the presence of CRH in the placenta of felids is novel and provides for the opportunity in future studies to investigate if CRH could be used as a molecular biomarker of early pregnancy detection in feline species of which there is not currently one. Ultrasonography can reliably detect a pregnancy at 25-35 days of gestation, whereas routine radiography not until ~42 days of gestation. An early pregnancy marker that could be used in the breeding management of endangered felid species would greatly benefit and increase efficiency and success in captive breeding programs. Urinary relaxin has been tested as a potential marker for early pregnancy in both domestic and wild cats (16, 17) but was only able to be detected in maternal serum at 25-30 days of gestation using a commercial ELISA assay (32). Subsequently, a radioimmunoassay was developed which successfully detected relaxin in urine samples between 21 and 28 days of pregnancy (16). Although maternal serum CRH levels from 2 early pregnant felids were not statistically higher (P > 0.05) at ~21 days of pregnancy, there was a ~24% increase in CRH serum levels compared to non-pregnant feline. Serial serum samples from queens starting at day of ovulation are needed to define the stage when CRH can be reliably detected in maternal blood and therefore used as a potential biomarker for the detection of early pregnancy in the queen. Finally, although ACTH also has been detected in human placentas and likely could be present in feline placentas, maternal levels do not nearly increase as much during pregnancy; at term reported levels are ~55pg/ml and ~4000pg/ml for ACTH and CRH (33).

In summary, this is the first report on the presence and localization of CRH in feline placenta. Furthermore, preliminary data indicates that CRH can be detected early in pregnancy in the maternal blood of queens using ELISA. This finding opens the door to future investigations to determine if CRH can be used as an early pregnancy biomarker for captive endangered feline species in ART breeding programs, and for further investigations into the physiological role of CRH in feline pregnancy.

## Data availability statement

The raw data supporting the conclusions of this article will be made available by the authors, without undue reservation.

## Ethics statement

This study uses tissues and blood samples obtained from a local animal shelter (Animal Friends Alliance, Fort Collins, CO). Colorado State University Institutional Animal Care & Use Committee did not require the study to be reviewed or approved by an ethics committee because these were samples opportunistically obtained after a routine surgical procedure was performed. These samples were disposed of after the surgery had taken place.

## Author contributions

MW performed the PCR and Western blot experiments and wrote the first draft of this manuscript. PM and EP conducted the ELISA and IHC experiments, respectively. GB and FH developed the idea and wrote the manuscript. GB provided direction and guidance on the molecular experiments, and FH coordinated and collected the tissues. All authors contributed to the article and approved the submitted version.
